# Reshaping Intratumoral Mononuclear Phagocytes with Antibody‐Opsonized Immunometabolic Nanoparticles

**DOI:** 10.1002/advs.202303298

**Published:** 2023-10-22

**Authors:** Chang Liu, Yanfeng Zhou, Daoxia Guo, Yan Huang, Xiaoyuan Ji, Qian Li, Nan Chen, Chunhai Fan, Haiyun Song

**Affiliations:** ^1^ State Key Laboratory of Oncogenes and Related Genes Center for Single‐Cell Omics School of Public Health Shanghai Jiao Tong University School of Medicine Shanghai 200025 China; ^2^ College of Chemistry and Materials Science The Education Ministry Key Lab of Resource Chemistry Joint International Research Laboratory of Resource Chemistry of Ministry of Education Shanghai Key Laboratory of Rare Earth Functional Materials and Shanghai Frontiers Science Center of Biomimetic Catalysis Shanghai Normal University Shanghai 200234 China; ^3^ School of Chemistry and Chemical Engineering Frontiers Science Center for Transformative Molecules and National Center for Translational Medicine Shanghai Jiao Tong University Shanghai 200240 China

**Keywords:** biomimetic nanoparticle, immunometabolic reshaping, innate immune stimulation, lipid metabolic reprogramming, mononuclear phagocyte

## Abstract

Mononuclear phagocytes (MPs) are vital components of host immune defenses against cancer. However, tumor‐infiltrating MPs often present tolerogenic and pro‐tumorigenic phenotypes via metabolic switching triggered by excessive lipid accumulation in solid tumors. Inspired by viral infection‐mediated MP modulation, here enveloped immunometabolic nanoparticles (immeNPs) are designed to co‐deliver a viral RNA analog and a fatty acid oxidation regulator for synergistic reshaping of intratumoral MPs. These immeNPs are camouflaged with cancer cell membranes for tumor homing and opsonized with anti‐CD163 antibodies for specific MP recognition and uptake. It is found that internalized immeNPs coordinate lipid metabolic reprogramming with innate immune stimulation, inducing M2‐to‐M1 macrophage repolarization and tolerogenic‐to‐immunogenic dendritic cell differentiation for cytotoxic T cell infiltration. The authors further demonstrate that the use of immeNPs confers susceptibility to anti‐PD‐1 therapy in immune checkpoint blockade‐resistant breast and ovarian tumors, and thereby provide a promising strategy to expand the potential of conventional immunotherapy.

## Graphical abstract

Antibody‐opsonized immunometabolic nanoparticles target intratumoral mononuclear phagocytes (MPs) for binary lipid metabolic reprogramming and innate immune stimulation, reverse their immunosuppressive phenotypes to reboot immune surveillance in lipid‐enriched tumor microenvironment, and overcome immunotherapy resistance in breast and ovarian cancers.

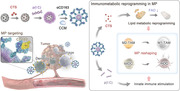

## 1 Introduction

Immunotherapy has provided revolutionary strategies for cancer treatment. However, many types of cancers develop immune escape, leading to unresponsiveness to immunomodulatory approaches such as immune checkpoint blockade (ICB).^[^ ^1^, ^2^, ^3^ ^]^ The failure in immune surveillance can be largely attributed to the dormancy of tumor‐infiltrating mononuclear phagocytes (MPs).^[^ ^4^, ^5^ ^]^ MPs are comprised of tissue‐resident macrophages and dendritic cells (DCs) as well as circulating monocytes, playing fundamental roles in immune surveillance and defense against tumors.^[^ ^6^, ^7^, ^8^, ^9^ ^]^ The classically activated macrophages in the tumor tissue, known as the M1‐like tumor‐associated macrophages (M1‐TAMs), exert tumoricidal functions through the secretion of pro‐inflammatory cytokines, generation of reactive oxygen/nitrogen species, and direct engulfment of cancer cells.^[^ ^10^, ^11^, ^12^ ^]^ DCs and macrophages can capture, process, and present tumor‐specific antigens to naïve resting T cells, thereby priming anti‐tumor T cell responses.^[^ ^13^, ^14^, ^15^ ^]^ In addition, cytosolic sensing of cancer cell‐derived DNA by MPs induces the production of type I interferons (IFNs) that inhibit tumor growth in direct and indirect ways.^[^ ^16^, ^17^ ^]^ Therefore, the restoration of MP immunocompetence is a prerequisite to yield favorable tumor responses and overcome immunotherapy resistance.

Metabolic abnormalities in the tumor microenvironment (TME) constitute a main obstacle to the anti‐tumor immunity of MPs.^[^ ^18^, ^19^, ^20^ ^]^ Recent studies reveal that many types of solid tumors contain high levels of lipid metabolites.^[^ ^21^, ^22^, ^23^ ^]^ This phenomenon is widely observed in breast, ovarian, colon, prostate, gastric, and melanoma skin cancers.^[^ ^24^, ^25^, ^26^ ^]^ It is becoming clear that increased lipid accumulation tilts the MPs from glycolysis to mitochondrial fatty acid oxidation (FAO) as a primary energy source. As a consequence, tumor‐infiltrating DCs differentiate toward a tolerogenic phenotype (tolDCs), displaying low antigen‐presenting capacity and poor T cell priming.^[^ ^27^, ^28^, ^29^ ^]^ The TAMs are polarized into the M2‐like subsets (M2‐TAMs) that suppress inflammation and T cell functions.^[^ ^30^, ^31^ ^]^ Accordingly, MP‐targeted lipid metabolic reprogramming represents a tempting strategy to reboot immune activation and potentiate the efficacy of cancer treatment.

Nanoparticles (NPs) delivering pathogen signals have been applied to immunomodulation in various cancer models.^[^ ^32^, ^33^, ^34^, ^35^ ^]^ However, the potential of fabricating NPs into pathogen mimics that possess better biomimetic interfaces for more comprehensive reshaping of tumor‐educated immune cells is largely unexplored. More than 35 types of viruses can infect MPs and drive immune responses and metabolic switching.^[^ ^36^, ^37^, ^38^ ^]^ Initial virus‐MP interaction requires the attachment of virus particles to specific cell surface receptors. Opsonization of virus particles by antibody or complement confers a convenient route for viral entry. For example, the antibody‐opsonized SARS‐CoV‐2 virus can infect monocytes and macrophages via the IgG Fc receptor CD16 and subsequently induce viral RNA‐dependent IFN production and metabolic changes.^[^ ^39^, ^40^, ^41^ ^]^ Inspired by the process of virus‐mediated MP modulation, we design a type of antibody‐opsonized immunometabolic NPs (immeNPs) for targeted dual interventions in tumor‐infiltrating MPs. We find that the binary metabolic and immune regulation of MPs strongly elicits the secretion of pro‐inflammatory cytokines and the recruitment of CD8^+^ T cells to the TME, and more importantly, renders ICB‐resistant cancers susceptible to anti‐PD‐1 (αPD‐1) therapy. As proofs of concept, combined treatment with the immeNPs and αPD‐1 triggers robust innate and adaptive immunity to restrain tumor growth in orthotopic 4T1 triple‐negative breast cancer (TNBC) and ID8 ovarian cancer, which opens up a new avenue for cancer immunotherapy.

## 2 Results

### 2.1 Design, Fabrication, and Characterization of immeNPs

**Figure 1:**
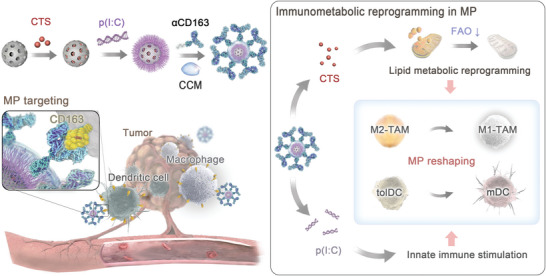
Schematic diagram for immeNP‐mediated immunometabolic reshaping of tumor‐infiltrating MPs. The antibody‐opsonized immeNPs specifically target the CD163^+^ tumor‐infiltrating TAMs and DCs for the delivery of FAO regulator CTS and viral RNA analog p(I:C). CTS‐mediated lipid metabolic reprogramming coordinates with p(I:C)‐stimulated antiviral response to amplify innate immunity, and subsequently induce M2 to M1 TAM repolarization, tolerogenic to mature DC differentiation, and cytotoxic T cell recruitment for superior therapeutic outcomes in murine models of orthotopic TNBC and ovarian cancer.

We designed a type of immeNPs to rehabilitate the anti‐tumor immunity of lipid‐laden MPs via binary metabolic and immune modulation. The immeNPs utilized cancer cell membrane (CCM) camouflage for tumor homing and antibody‐receptor interaction for specific anchoring to the CD163 marker on the surfaces of M2‐TAMs and DCs. The immeNP inner cores employed mesoporous silica nanoparticles (MSNs) for sequential loading of the FAO regulator cryptotanshinone (CTS) and the viral RNA analog polyinosinic‐polycytidylic acid (referred to as p(I:C) hereafter). Upon immeNP internalization, the MPs underwent lipid metabolism reprogramming via CTS‐mediated FAO inhibition, and innate immune stimulation in parallel via p(I:C)‐induced antiviral response, thereby synergistically promoting M2‐to‐M1 TAM repolarization and tolDC‐to‐mature DC (mDC) differentiation (**Figure** **1**).

**Figure 2:**
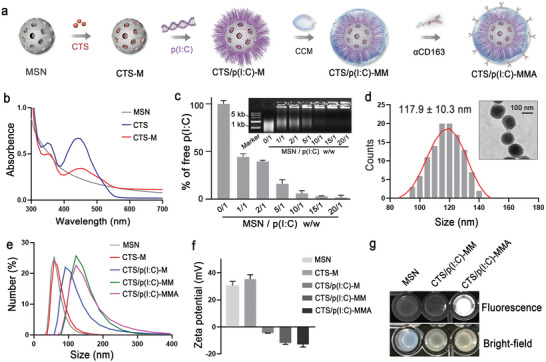
immeNP fabrication and characterization. a) Schematic design of immeNP assembly. b) The UV–vis absorption spectra of unloaded MSNs, free CTS, and CTS‐loaded MSNs (CTS‐M). c) The gel retardation electrophoresis and quantitative analysis of free p(I:C) levels after nanoparticle adsorption under the condition of various nanoparticle to p(I:C) mass ratios. d) The size distribution of CTS/p(I:C)‐MMA in TEM. Over 110 particles were counted. Insert TEM image of CTS/p(I:C)‐MMA. e) The DLS analysis for various forms of nanoparticles. f) The zeta potentials of indicated nanoparticles. g) The images of unloaded MSNs, CTS/p(I:C)‐MM and fluorescent antibody‐coated CTS/p(I:C)‐MMA under visible light or 405 nm laser irradiation. Data are represented as mean ± SD (*n* = 3) in (c,f).

The MSNs were fabricated according to a heterogeneous oil–water biphasic reaction approach, and amino groups were introduced via the modification with 3‐aminopropyltriethoxysilane (APTES).^[^ ^42^, ^43^, ^44^ ^]^ For immeNP assembly, the MSNs were loaded with CTS (CTS‐M) and subsequently with p(I:C) (CTS/p(I:C)‐M), coated with CCM from 4T1 or ID8 cells (CTS/p(I:C)‐MM), and modified with 1,2‐distearoyl‐sn‐glycero‐3‐phosphoethanolamine‐poly(ethylene glycol) (DSPE‐PEG) conjugated anti‐CD163 antibody (CTS/p(I:C)‐MMA) (**Figure** **2**a). The aminopropyl‐functionalized MSNs presented a spherical shape with a uniform particle size of 65.7 ± 7.2 nm under the transmission electron microscope (TEM) (Figure S1, Supporting Information). CTS showed a characteristic absorption peak at 450 nm, which enabled its quantification after binding to the MSNs with a maximal loading efficiency of 25.2 ± 1.2% (Figure 2b and Figure S2, Supporting Information). Next, anionic p(I:C) molecules were loaded to the CTS‐M surface via electrostatic adsorption, which could be monitored by the agarose gel electrophoresis (Figure 2c). The resultant CTS/p(I:C)‐M could efficiently protect p(I:C) from nuclease degradation and substantially improve p(I:C) stability (Figure S3, Supporting Information). After coating with CCM and opsonization with anti‐CD163, the assembled CTS/p(I:C)‐MMA exhibited an average particle size of 117.9 ± 10.3 nm in the TEM image (Figure 2d). The process of immeNP assembly was also characterized by the measurements of dynamic light scattering (DLS) and zeta potentials. The unloaded MSNs (70.4 ± 7.1 nm), CTS‐M (74.1 ± 6.4 nm), CTS/p(I:C)‐M (103.2 ± 2.5 nm), CTS/p(I:C)‐MM (119.5 ± 3.2 nm), and CTS/p(I:C)‐MMA (123.7 ± 5.9 nm) displayed stepwise increases in the hydrodynamic sizes (Figure 2e). Compared to the positively charged MSNs (30.4 ± 3.3 mV) and CTS‐M (35.3 ± 3.3 mV), the CTS/p(I:C)‐M were negatively charged (−4.5 ± 0.1 mV). After membrane coating and antibody decoration, the CTS/p(I:C)‐MMA showed a zeta potential value of −13.0 ± 1.9 mV (Figure 2f). Using a type of fluorophore‐labeled antibodies, we were able to confirm the successful immobilization of the DSPE‐PEG conjugated antibodies on the immeNPs, which produced intense fluorescence with laser irradiation at 405 nm wavelength (Figure 2g).

### 2.2 Binary Metabolic and Immune Regulation of MPs In Vitro

**Figure 3:**
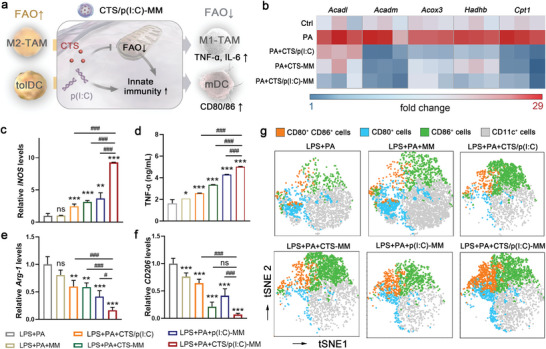
Binary MP modulation by immeNPs in vitro. a) A scheme for immunometabolic reshaping of TAMs and DCs by immeNPs. b) Expression heat map of the FAO‐related genes in LPS‐induced BMDMs after indicated treatment. c) RT‐PCR analysis of M1‐type marker *iNOS* expression and d) the secretion of inflammatory cytokine TNF‐α in the BMDMs after indicated treatment. e,) RT‐PCR analysis of M2‐type markers *Arg‐1* and f) *CD206* expression in the BMDMs after indicated treatment. g) The t‐SNE maps of CD80^+^, CD86^+^, CD80^+^CD86^+^, and other CD11c^+^ BMDCs are labeled blue, green, orange, and gray, respectively. Data are represented as mean ± SD (*n* = 3) in (c–f). One‐way ANOVA with Tukey's post‐hoc test, ns means not significant, ***p < 0.001, **p < 0.01, *p < 0.05, ^###^ *p* < 0.001, ^#^ *p* < 0.05.

First, we explored the in vitro efficacy of CTS/p(I:C)‐MM in the reshaping of lipid‐induced M2‐TAMs and tolDCs, since the anti‐CD163 modification mainly served for specific MP targeting in vivo. We employed CTS/p(I:C)‐MM at the concentration of 100 µg mL^−1^ MSNs, equivalent to 16.8 µg mL^−1^ CTS and 10 µg mL^−1^ p(I:C) that were both in recommended dose ranges for cell‐based assays.^[^ ^45^, ^46^ ^]^ We expected that CTS‐mediated FAO inhibition and p(I:C)‐mediated immune stimulation would act synergistically to promote M2‐to‐M1 macrophage repolarization and DC maturation in the lipid‐enriched environment (**Figure** **3**a). Lipopolysaccharide (LPS) stimulation of bone marrow‐derived macrophages (BMDMs) resulted in M1‐like polarization, which was characterized by elevated expression of inducible nitric oxide synthase (*iNOS*) and interleukin 12 (*IL‐12*), and increased secretion of inflammatory cytokines including tumor necrosis factor α (TNF‐α) and IL‐6 (Figure S4a–d, Supporting Information). In contrast, the expression levels of M2 macrophage markers arginase 1 (*Arg‐1*) and *CD206* were repressed by LPS stimulation (Figure S4e,f, Supporting Information). Exposure to palmitic acid (PA) during the LPS stimulation strongly inhibited the levels of the aforementioned M1 macrophage markers, while largely potentiated the expression of M2 phenotype markers (Figure S4, Supporting Information). These alterations in BMDMs were associated with enhanced FAO by exogenous PA, as revealed by increased expression levels of FAO‐related genes and enhanced FAO activities. Treatment with CTS‐MM or CTS/p(I:C)‐MM prevented PA‐induced upregulation in FAO‐related gene expression and enhancement of FAO activities (Figure 3b, and Figures S5 and S6a, Supporting Information).^[^ ^47^, ^48^, ^49^ ^]^ Consistent with the metabolic changes, CTS‐MM promoted the M1 phenotype markers (expressions of *iNOS* and *IL‐12*; secretion of TNF‐α and IL‐6) and repressed the M2 phenotype markers (*Arg‐1* and *CD206*) in PA‐treated BMDMs. Induction of antiviral response via p(I:C)‐MM displayed similar effects on M1/M2 polarization, and CTS/p(I:C)‐MM showed synergistic effects from combined metabolic reprogramming and innate immune stimulation (Figure 3c–f and Figure S7, Supporting Information).

Similar to our observation in BMDMs, incubation with PA activated the expression of FAO‐related genes and enhanced FAO activities in bone marrow‐derived DCs (BMDCs), which could be reversed by CTS‐MM or CTS/p(I:C)‐MM (Figures S6b and S8, Supporting Information). As a result, LPS‐stimulated DC maturation, marked by the presence of co‐stimulatory molecules CD80 and CD86 on the cell surface, was abolished upon PA exposure (Figure S9, Supporting Information). Treatment with CTS‐MM, p(I:C)‐MM or CTS/p(I:C)‐MM potentiated BMDC maturation in the PA‐enriched environment, with the combination treatment eliciting the highest levels of CD80 and CD86 on the BMDC surfaces (Figure 3g and Figure S10, Supporting Information). These results suggested that the immeNP‐mediated binary metabolic and immune regulation could reprogram lipid‐induced M2‐TAMs and tolDCs into their anti‐tumor phenotypes via parallel FAO inhibition and innate sensing.

### 2.3 immeNPs Enable ICB Therapy in Orthotopic TNBC

Cell targeting poses a major challenge for efficient in vivo delivery. The CD163 scavenger receptor was reported as a cell surface marker of M2‐TAMs.^[^ ^50^, ^51^, ^52^ ^]^ We found it abundantly expressed in BMDMs, BMDCs, and Raw264.7 macrophages, but poorly expressed in 4T1 and Hepa1‐6 cancer cells as well as NIH/3T3 fibroblasts (Figure S11a, Supporting Information). The anti‐CD163 antibody‐opsonized immeNPs, assembled from fluorescein (FAM)‐incorporated MSNs (CTS/p(I:C)‐M^FAM^MA), exhibited significantly higher uptake efficiency in the Raw264.7 macrophages than their undecorated version (CTS/p(I:C)‐M^FAM^M) (Figure S11b, Supporting Information). In the mice bearing orthotopic 4T1 TNBC, we observed that the tumor‐infiltrating TAMs and DCs prominently upregulated their *CD163* expression compared to other cells in the tumor tissue (Figure S12a, Supporting Information). Accordingly, intravenously injected CTS/p(I:C)‐M^FAM^MA displayed much better co‐localization efficiency with TAMs than the undecorated CTS/p(I:C)‐M^FAM^M, confirming their targeting specificity (Figure S12b, Supporting Information). Moreover, the in vivo localization kinetics studies for the immeNPs were conducted with near‐infrared fluorescent dye Cy5.5‐labeled CTS/p(I:C)‐M^Cy5.5^ and CTS/p(I:C)‐M^Cy5.5^MA. Both CTS/p(I:C)‐M^Cy5.5^ and CTS/p(I:C)‐M^Cy5.5^MA accumulated in the tumors through intravenous injection, whereas CTS/p(I:C)‐M^Cy5.5^MA displayed much stronger fluorescence signals in the tumors than CTS/p(I:C)‐M^Cy5.5^ at 24 h post‐injection (Figure S13a, Supporting Information). The quantifications of ex vivo fluorescence imaging from major organs and tumors confirmed that cancer cell membrane coating in combination with antibody opsonization preferentially enhanced the accumulation of immeNPs in the tumors, but not in the main organs such as liver, spleen, lung, or lymph nodes (Figure S13b, Supporting Information).

Therefore, we utilized anti‐CD163 modified immeNPs for in vivo study. We assessed the anti‐tumor activities of CTS/p(I:C)‐MMA in the orthotopic 4T1 TNBC model. Compared to the control mice, combined administration of free CTS and p(I:C) (CTS/p(I:C)) only mildly delayed tumor growth, whereas CTS/p(I:C)‐MMA (5 doses, administered every 3 days) displayed more potent inhibitory effect (Figure S14a,b, Supporting Information). Consistently, treatment with CTS/p(I:C)‐MMA was more efficient than free CTS/p(I:C) in decreasing the levels of tumor‐infiltrating M2‐TAMs and recruiting CD8^+^ T cells (Figure S14c,d, Supporting Information). To determine whether the anti‐tumor effects of immeNPs depended on MPs, we utilized clodronate liposomes to deplete macrophages and DCs in the TNBC‐bearing mice during CTS/p(I:C)‐MMA treatment.^[^ ^53^, ^54^ ^]^ Consistent with previous studies, clodronate showed a mild inhibitory effect on tumor growth, suggesting the pro‐tumor activities of MPs in the immunosuppressive TME.^[^ ^55^ ^]^ The immeNPs could not further restrict tumor growth in clodronate‐treated mice, indicating that the anti‐tumor effects of CTS/p(I:C)‐MMA relied on MP reprogramming (Figure S15, Supporting Information).

**Figure 4:**
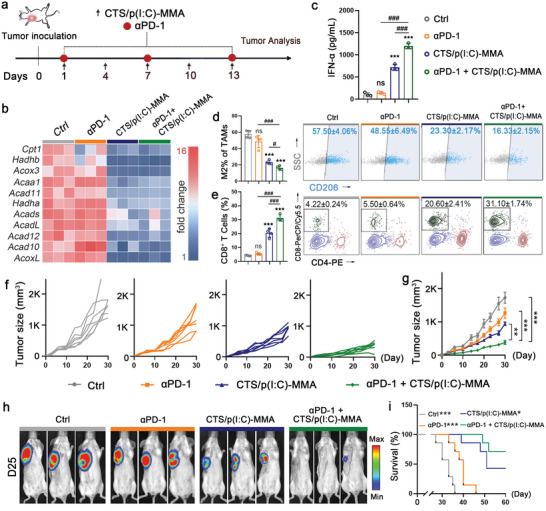
immeNPs enable ICB therapy in orthotopic 4T1 TNBC. a) Schematic outline of experimental design for combination therapy. b) Expression heat map of the FAO‐related genes (*n* = 3). c) Levels of serum IFN‐α in tumor‐bearing mice (*n* = 3). d) Flow cytometric analysis of the percentage of M2‐TAM population in TAMs (gated on CD45^+^CD11b^+^F4/80^+^) (n = 4). e) Flow cytometric analysis of the percentage of CD8^+^ T cells in total immune cells (gated on CD45^+^ cells) within tumors (*n* = 4). f) Individual tumor growth kinetics (*n* = 7). g) Average tumor growth curves (*n* = 7). h) Representative bioluminescence images of mice on day 25. See also Figure S17, Supporting Information for complete data at various time points. i) Animal survival curves (*n* = 7). Data are represented as mean ± SD. One‐way ANOVA with Tukey's post‐hoc test for (c–e,g), Log‐rank (Mantel–Cox) test for (i). ns means not significant, ***p < 0.001, **p < 0.01, *p < 0.05, ^###^ *p* < 0.001, ^#^ *p* < 0.05.

The 4T1 tumor is poorly immunogenic, which impedes its accessibility by CD8^+^ T cells and response to ICB therapy.^[^ ^56^, ^57^, ^58^, ^59^ ^]^ Given the capacities of the immeNPs in MP remodeling and CD8^+^ T cell recruitment, we continued to investigate whether combined treatment with αPD‐1 and the immeNPs could overcome ICB resistance in the orthotopic TNBC model (**Figure** **4**a). Compared to the control or αPD‐1 treatment, monotherapy with CTS/p(I:C)‐MMA and combination therapy with αPD‐1 and CTS/p(I:C)‐MMA largely suppressed the expression of FAO‐related genes to similar levels (Figure 4b and Figure S16, Supporting Information). To evaluate the effects of combination therapy on DC maturation, we measured the levels of IFN‐α produced by mDCs. αPD‐1 treatment had a neglectable effect on IFN‐α secretion by itself. In contrast, CTS/p(I:C)‐MMA markedly induced IFN‐α secretion and the combination therapy further promoted DC maturation (Figure 4c). Similarly, the combination of CTS/p(I:C)‐MMA and αPD‐1 could significantly potentiate the immeNP‐mediated M2‐TAM repolarization and CD8^+^ T cell infiltration (Figure 4d,e). Although the TNBC tumors were resistant to αPD‐1 treatment, combined treatment with αPD‐1 and the immeNPs elicited synergistic effects on the inhibition of tumor growth (Figure 4f,g). The effectiveness of the combination therapy was also confirmed by the in vivo bioluminescent imaging. While the αPD‐1 treatment failed to prevent tumor cell expansion, its combination with the immeNPs robustly weakened the intensities of bioluminescent signals (Figure 4h and Figure S17, Supporting Information). Moreover, the combined treatment displayed beneficial effects on the survival rates. The control mice had a median survival time of 33 days. In comparison, αPD‐1 treatment and immeNP treatment extended the median survival time to 40 days and 51 days, respectively. Five out of seven mice receiving the combination therapy survived over 60 days (Figure 4i). To investigate the therapeutic effects of immeNPs on larger tumors, we initiated immeNP administration 10 days after tumor cell inoculation (≈200 mm^3^ in tumor sizes). The immeNPs alone or their combination with αPD‐1 still restrained tumor growth effectively, manifesting that the anti‐tumor efficacy of the immeNPs was not noticeably affected by the starting time point of administration (Figure S18, Supporting Information).

To examine the safety of the CTS/p(I:C)‐MMA‐based combination immunotherapy, the major organs and blood of the mice were collected for histological analysis and blood biochemistry analysis after treatment. Hematoxylin and eosin (H&E) staining of major organ sections revealed no significant morphological damage (Figure S19, Supporting Information). The measurement of serum markers of liver and kidney functions also suggested that the use of CTS/p(I:C)‐MMA in combination with αPD‐1 therapy did not cause noticeable side effects at the administered doses (Figure S20, Supporting Information).

### 2.4 immeNPs Sensitize Orthotopic Ovarian Cancer to ICB

**Figure 5:**
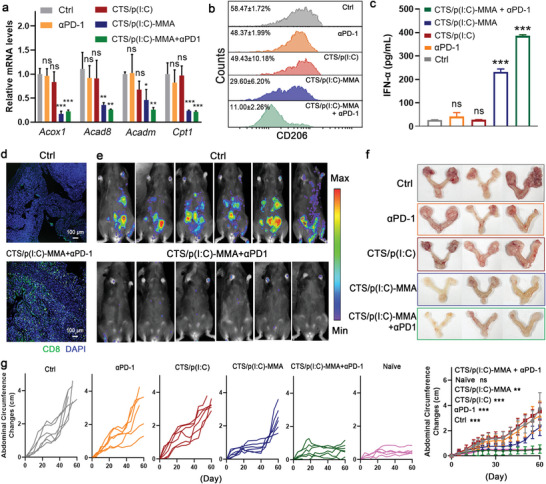
immeNPs sensitize orthotopic ID8 ovarian cancer to ICB. a) RT‐PCR analysis of the FAO‐related genes (*n* = 3). b) Flow cytometric analysis of the percentage of M2‐TAM population in TAMs (gated on CD45^+^CD11b^+^F4/80^+^) (*n* = 3). c) Levels of serum IFN‐α in mice after indicated treatment (*n* = 3). d) Representative immunofluorescence images of CD8^+^ T cells in tumor sections. See also Figure S23, Supporting Information for complete data. Scale bars, 100 µm. e) The in vivo bioluminescence images of mice day 30. See also Figure S24, Supporting Information for complete data. f) The images of tumor ovaries on day 60. g) Individual and average abdominal circumference changes in the tumor‐bearing mice and healthy mice (naïve) (*n* = 6). Data are represented as mean ± SD in (a−c,g). One‐way ANOVA with Tukey's post‐hoc test, ns means not significant, ***p < 0.001, **p < 0.01, *p < 0.05.

The applicability of the immeNPs in ICB‐resistant tumors was further tested in an orthotopic ID8 ovarian cancer model. Metabolic regulation via CTS/p(I:C)‐MMA alone or the combined treatment significantly decreased the expression of FAO‐related genes in ID8 tumors (**Figure** **5**a). The ratios of M2‐TAMs in the tumor‐infiltrating TAM population were prominently reduced in the mice receiving immeNP treatment, and this effect was further strengthened when combined with αPD‐1 therapy (Figure 5b and Figure S21, Supporting Information). Likewise, the combination therapy boosted immeNP‐mediated DC maturation, as indicated by the levels of secreted IFN‐α and cell surface CD86 (Figure 5c and Figure S22, Supporting Information). Consistent with its high efficiency in MP reshaping, the combined treatment by CTS/p(I:C)‐MMA and αPD‐1 elicited CD8^+^ T cell recruitment more potently than other treatments (Figure 5d and Figure S23, Supporting Information). The levels of innate and adaptive immune activation were closely correlated to the efficacy of ovary tumor inhibition. As revealed by in vivo bioluminescence imaging and resected ovaries, αPD‐1 or CTS/p(I:C)‐MMA monotherapy showed mild to moderate effects on reducing the tumor burden, and the combination therapy eliminated the cancer cells from the ovaries (Figure 5e,f and Figure S24, Supporting Information). We also measured the changes in abdominal circumferences and the levels of serum CA‐125, two parameters associated with ovarian cancer progression. αPD‐1 treatment alone showed no effect on abdominal circumference increases or circulating CA‐125 levels. The immeNPs noticeably diminished the changes in abdominal circumferences and the levels of serum CA‐125, both of which were further suppressed by the combined treatment (Figure 5g and Figure S25, Supporting Information). Together, these results indicated that the immeNP‐mediated MP remodeling efficiently switched ICB‐refractory TNBC and ovarian cancer to ICB‐sensitive phenotypes, and synergized with αPD‐1 treatment for superior therapeutic outcomes.

## 3 Discussion

The process of MP reshaping by the immeNPs mimicked virus‐MP interaction in several aspects. First, binding to cell surface receptors facilitated the recognition of MPs. Second, lipid metabolic reprogramming altered the immunosensitivities of MPs. Lastly, the cytosolic release of viral RNA analog drove immune responses. Combining these features, the immeNPs rebooted the anti‐tumor immunity of MPs in the lipid‐enriched TME. Conventional immunotherapy usually relies on delivering pathogen‐associated immune stimuli (e.g., bacterial‐ or virus‐related molecules) or releasing an “immune brake” on effector T cells (via immune checkpoint inhibitors), whereas dysfunctions in lipid‐laden MPs render them unable to sense external immune stimuli or recruit effector T cells for tumor infiltration, leading to immunotherapy resistance.^[^ ^28^, ^31^ ^]^ In contrast, the immeNPs can rectify lipid metabolic abnormalities in tumor‐infiltrating MPs and reverse their immunosuppressive activities to reinstate efficient immune surveillance. Given lipid accumulation is detected in a broad variety of solid tumors, the concept of immeNPs may represent a generalized strategy to prevent immune evasion and overcome ICB resistance.

In summary, we designed MP‐targeting immeNPs, which reshaped tumor‐infiltrating M2‐TAMs and tolDCs via binary metabolic and immune regulation. The antibody‐opsonized immeNPs were preferentially internalized by MPs, delivering FAO regulator and viral RNA analog for parallel lipid metabolic reprogramming and innate immune stimulation. Consequently, immeNP treatment induced M2‐to‐M1 TAM repolarization and tolDC‐to‐mDC differentiation, and promoted cytotoxic T cell infiltration in low immunogenic breast and ovary tumors. Importantly, immunometabolic remodeling of TME by the immeNPs tilted these ICB‐resistant tumors toward an ICB‐sensitive phenotype. Combined treatment with the immeNPs and αPD‐1 produced synergistic effects on the suppression of tumor progression in 4T1 TNBC and ID8 ovary cancers. The immeNP‐mediated dual reshaping of tumor‐infiltrating MPs offers an innovative way to broaden the applicability of cancer immunotherapy.

## 4 Experimental Section

### Preparation of CTS/p(I:C)‐M

To synthesize the MSN core, hexadecyltrimethylammonium bromide (CTAB, 1.82 g) and NH_4_F (0.5 g) were mixed and stirred in an oil bath at 80 °C. After 1 h stirring, tetraethyl orthosilicate (TEOS, 6 mL) was added dropwise into the solution, followed by a 2 h hydrolysis/condensation reaction to form MSNs. The MSNs were collected by centrifugation (10 000 rpm, 15 min) and washed with water and pure ethanol. For the preparation of CTS/p(I:C)‐M, CTS (MedChemExpress, HY‐N0174) was added into the MSN solution and stirred for 1 h at room temperature. Afterward, p(I:C) (InvivoGen, tlrl‐pic) was added dropwise and stirred for 6 h. The mixture was centrifuged at 8000 g for 10 min to collect CTS/p(I:C)‐M, followed by gentle washing with ultrapure water twice to remove free CTS and p(I:C). The levels of free p(I:C) after adsorption, with different MSNs to p(I:C) mass ratios, were examined by agarose gel electrophoresis at 100 V. After staining with Gel‐Red (Beyotime, D0140), the gel imaging was performed under UV irradiation and analyzed by the Image J software. To test the stability of p(I:C) in CTS/p(I:C)‐M, free p(I:C) or CTS/p(I:C)‐M were incubated with 10% serum (48 h), or 1 mU RNase A (2 h) at 37 °C. Thereafter, the samples were examined by agarose gel electrophoresis and the data were analyzed by the Image J software.

### Preparation of CTS/p(I:C)‐MMA and Nanoparticle Characterization

CCM and CCM‐coated nanoparticles were obtained as previously described.^[^ ^60^, ^61^, ^62^ ^]^ Briefly, 4T1 or ID8 cells were cultured and harvested. The cancer cells were washed three times with phosphate‐buffered saline (PBS) and ground in low permeability buffer containing EDTA‐free protease inhibitors (Thermo Scientific, A32955). The nuclei were removed by centrifugation at 5000 g, 4 °C for 5 min. The supernatant was centrifuged at 150 000 g, 4 °C for 1 h. The cell membrane was collected and extruded 15 times through a 400 nm polycarbonate porous membrane with an Avanti mini‐extruder. To prepare CTS/p(I:C)‐MM, the obtained CCM was mixed with CTS/p(I:C)‐M in 20 µL Milli‐Q water, sonicated for 5 min, incubated at 4 °C overnight, and washed three times with water to remove free CCM. For preparation of CTS/p(I:C)‐MMA, DSPE‐PEG conjugated anti‐CD163 antibody (DSPE‐PEG‐αCD163) was synthesized using 1‐(3‐dimethylaminopropyl)−3‐ethylcarbodiimide hydrochloride (EDC) (Sigma,25952‐53‐8)/N‐hydroxysuccinimide (NHS) (Sigma, 6066‐82‐6). Briefly, DSPE‐PEG‐COOH (Ruixibio) was activated by 100 µL NHS (0.6 µg mL^−1^) and 100 µL EDC (0.23 µg mL^−1^) before incubating with αCD163 (Abcam, ab182422) for 2–4 h. The mixture was incubated with 500 µL CTS/p(I:C)‐MM (1 mg mL^−1^) for another 2–4 h, and was centrifuged at 5000 g, 4 ˚C for 15 min to remove excessive EDC/NHS and unconjugated αCD163. The Alexa Fluor 488‐conjugated IgG (Abcam, ab150113) was used in parallel to confirm the immobilization of antibodies on CTS/p(I:C)‐MM.

A ZEN3690 Zetasizer particle analyzer (Malvern) was used for the DLS measurement of different forms of nanoparticles. For TEM analysis, samples were added to carbon‐coated 400 square mesh copper grids for drying. The grids were negatively stained with 1% uranyl acetate for TEM imaging (Hitachi), which were further analyzed by the Nano Measurer software. The average particle size was determined by measuring the diameters of 80 particles (MSNs) and 110 particles (CTS/p(I:C)‐MMA) in the TEM image.

### Cell Culture

The mouse 4T1 TNBC cell line was obtained from the Cell Bank of the Chinese Academy of Sciences (Shanghai). The mouse ID8 ovarian cancer cells were obtained from Fuheng Biology Science and Technology Co., Ltd. (Shanghai). The 4T1 and ID8 cells were transfected with recombinant lentivirus for stable expression of the firefly luciferase (fLuc) and were cultured in the Roswell Park Memorial Institute (RPMI) 1640 medium and Dulbecco's modified Eagle medium (DMEM), respectively, both of which were supplemented with 10% fetal bovine serum (FBS) and 1% penicillin/streptomycin.

Bone marrow cells sorted from C57BL/6 mice were cultured in the DMEM containing GM‐CSF (20 ng mL^−1^) and IL‐4 (10 ng mL^−1^) for 5 days to obtain BMDMs (adherent cells) and BMDCs (non‐adherent cells). Half of the medium was changed on day 3. BMDMs and BMDCs were seeded in 24‐well plates at a density of 1 × 10^6^ cells per well for treatment. To stimulate FAO, 25 µM bovine serum albumin (BSA)‐conjugated palmitate (Sigma) was added to the cell culture. LPS‐induced BMDMs or BMDCs were cultured in standard or PA‐loaded medium and were incubated with unloaded MM (MSNs: 100 µg mL^−1^), CTS‐MM (MSNs: 100 µg mL^−1^; CTS: 16.8 µg mL^−1^), p(I:C)‐MM (MSNs: 100 µg mL^−1^; p(I:C): 10 µg mL^−1^), CTS/p(I:C)‐MM (MSNs: 100 µg mL^−1^; CTS: 16.8 µg mL^−1^; p(I:C): 10 µg mL^−1^) or free CTS/p(I:C) of corresponding concentrations.

### Enzyme‐Linked Immunosorbent Assay (ELISA)

Secretion of cytokines (TNF‐α and IL‐6) in the supernatant and the intracellular FAO activities were measured using the TNF‐α ELISA kit (Boster, EK0527), the IL‐6 ELISA kit (Boster, EK0411), and the FAO ELISA kit (SINOBESTBIO, YX‐060115 M) according to the manufacturer's instruction. To measure the levels of IFN‐α and CA125 in mouse serum, 1 mL venous blood was harvested and centrifuged immediately at 3000 rpm for 20 min. The supernatant was collected and measured with the mouse IFN‐α ELISA assay kit (Invitrogen, BMS6027) or the mouse CA125 ELISA kit (Jianglaibio, JL10792).

### Real‐Time PCR

Total RNA from harvested cells was isolated by TriZol (Ambion) and reverse‐transcribed into complementary DNA with the ReverTraAce qPCR RT Kit (TOYOBO). Quantitative real‐time PCR was performed with the SYBR Green Realtime PCR Master Mix (TOYOBO) in the StepOne Real‐Time PCR System (Applied Biosystems). The relative expression levels of *CD163*, *iNOS*, *IL‐12*, *Arg‐1*, *CD206*, and FAO‐related genes were normalized to that of the control gene *GAPDH*.

### Animal Treatment

Cultured 4T1 or ID‐8 tumor cells were trypsinized, centrifuged (800 rpm, 3 min), and washed twice in PBS. The orthotopic TNBC model was generated by injecting 1 × 10^5^ fLuc‐4T1 cells (in 50 µL matrigel) into the mammary fat pad of 8‐week‐old female Balb/c mice (day 0). Mock PBS, CTS/p(I:C)‐MMA (1 mg MSNs, 168 µg CTS and 100 µg p(I:C) per mouse), free CTS or CTS/p(I:C) was injected intravenously on days 1, 4, 7, 10, and 13. αPD‐1 (Biolegend, 114111) (200 µg per mouse) was administered on days 1, 7, and 13. Tumor growth was monitored by a vernier caliper, and the tumor volume was calculated with the formula (volume = 0.5 × length × width^2^). Bioluminescence signals from tumor‐bearing mice were observed on days 5, 15, and 25. Mice were imaged under the IVIS spectrum in vivo imaging system (Perkin Elmer) 10 min after intraperitoneal injection of D‐Luciferin (150 µg g^−1^, Genomeditech, GM‐040611). On day 15, tumors were collected for FACS analysis. On day 30, major organs were isolated for H&E staining. To investigate whether the immeNPs could restrain larger tumors, mock PBS or CTS/p(I:C)‐MMA was injected intravenously on days 10, 13, 16, 19, and 22, and αPD‐1 was administered on days 10, 16, and 22.

To determine whether the anti‐tumor effects of immeNPs depended on MPs, clodronate liposomes were utilized to deplete macrophages and DCs in the TNBC‐bearing mice during CTS/p(I:C)‐MMA treatment. After tumor cell inoculation, mice were injected intraperitoneally with initial 150 µL clodronate liposomes (4 mg mL^−1^) or PBS liposomes on day 1. Mice were then treated with 75 µL liposomes every 4 days until the endpoint. CTS/p(I:C)‐MMA (1 mg MSNs, 168 µg CTS, and 100 µg p(I:C) per mouse) was injected intravenously on days 1, 4, 7, 10, and 13.

The ovarian cancer model was generated by intraperitoneally injecting 5 × 10^6^ fLuc‐ID8 cells (in 100 µL PBS) into C57BL/6 mice (day 0). Mock PBS, free CTS/p(I:C), or CTS/p(I:C)‐MMA (1 mg MSNs, 168 µg CTS, and 100 µg p(I:C) per mouse) was injected intraperitoneally on days 5, 9, 13, 17, 21, and 25. αPD‐1 (200 µg per mouse) was administered on days 5, 13, and 21. Tumor progression was monitored by the increase in abdominal circumference due to the accumulation of peritoneal ascites. Mice were euthanized when the abdominal circumference was ≥ 10 cm or when they exhibited reduced body condition due to tumor progression. Bioluminescence signals from tumor‐bearing mice were observed on days 10 and 30. For immunofluorescence imaging and FACS analysis, the ovarian tumors were collected on day 26. All mouse experiments were conducted following protocols approved by the Animal Care and Use Committee of Shanghai Jiao Tong University School of Medicine (A‐2022‐115).

### Ex Vivo Fluorescence Imaging

When the tumor sizes reached ≈300 mm^3^, CTS/p(I:C)‐M^Cy5.5^ or CTS/p(I:C)‐M^Cy5.5^MA (1 mg MSNs, 168 µg CTS, 100 µg p(I:C), and 20 µg Cy5.5 per mouse) was injected intravenously. The tumors, lymph nodes, and major organs of mice were dissected and imaged by an in vivo imaging system (PerkinElmer, IVIS spectrum CT) at 24 h post‐injection.

### Targeted Uptake of Antibody‐Opsonized immeNPs

The Raw 264.7 macrophages were incubated with CTS/p(I:C)‐M^FAM^M or CTS/p(I:C)‐M^FAM^MA for 90 min. The intensities of intracellular FAM signals were determined by imaging with a confocal laser scanning microscope (Leica). In vivo targeting of antibody‐opsonized immeNPs was evaluated in the 4T1 tumor model. 1 × 10^5^ 4T1 cells (in 50 µL matrigel) were injected into the mammary fat pad of 8‐week‐old female Balb/c mice (day 0). CTS/p(I:C)‐M^FAM^M or CTS/p(I:C)‐M^FAM^MA was injected intravenously on days 14, 15, and 16. The tumors were harvested on day 17. For analysis of the co‐localization between the immeNPs and TAMs, the tumor sections were stained with anti‐CD206 (1:400, Servicebio, GB113497) and Cy3‐conjugated secondary antibody (Servicebio, GB21301), followed by imaging with confocal laser scanning microscope (Leica) and quantification with the ImageJ software.

### Immunofluorescence Staining

Solid tumor nodules and surrounding tissues were excised from the ovaries and fixed with a neutral formalin solution (Servicebio, G1101) on day 26. Tumor sections were stained with anti‐CD8 beta (1:500, Servicebio, GB111742), anti‐CD86 (1:200, Abcam, ab119857), and fluorescent secondary antibodies, and imaged with confocal laser scanning microscope (Leica). The fluorescent intensities were measured with the Image J software.

### Flow Cytometry

Tumors were homogenized in PBS to obtain single‐cell suspensions, incubated with appropriate antibodies for measurement on a BD FACSAria cell sorter, and analyzed by the FlowJo software (Tree Star). APC/Cy7‐conjugated anti‐CD45 (BioLegend, 47045182), PE‐conjugated anti‐F4/80 (BioLegend, 123110), PerCP/Cy5.5‐conjugated anti‐CD11b (BioLegend, 101228), and BV421‐conjugated anti‐CD206 (BioLegend, 141717) were used to sort M2‐TAMs. Anti‐CD45‐APC/Cy7 (eBioscience, 47045182), anti‐CD4‐PE (Biolegend, 100408), and anti‐CD8‐PerCP/Cy5.5 (BD, 551162) were used to separate cytotoxic T lymphocytes (CD45^+^CD8^+^). The maturation of cultured BMDCs was verified with anti‐CD11c‐PE (Biolegend, 117308), anti‐CD80‐PerCP/Cy5.5 (Biolegend, 104721), and anti‐CD86‐BV421 (Biolegend, 105031).

### Analysis of Liver and Kidney Functions

To detect the serum markers of liver and kidney functions, 1 mL of venous blood was harvested from the mice with indicated treatment. After 20 min centrifugation at 3000 rpm, the supernatant was collected and the levels of alanine transaminase (ALT), aspartate transaminase (AST), creatinine (Cre), and blood urea nitrogen (BUN) in serum were measured with commercial assay kits (ALT: Nanjing Jiancheng, C00921; AST: Nanjing Jiancheng, C01021; Cre: Geruisi‐bio, G1204W; BUN: Geruisi‐bio, G1201W).

### Statistical Analysis

Statistical analysis was performed with GraphPad Prism. Data are shown as mean ± SD or mean ± SEM. Quantification of free p(I:C) levels in gel retardation assay (*n* = 3); Zeta potential analysis for various forms of nanoparticles (*n* = 3); Q‐PCR analysis of FAO‐related genes, M1‐ and M2‐type markers (*n* ≥ 3); Quantification of cytokines TNF‐α and IFN‐α (*n* = 3); ratios of M2‐TAM and CD8^+^ T cells (*n* ≥ 3); Average tumor growth curves, bioluminescence images, and survival curves of 4T1 tumor‐bearing mice (*n* = 7); Bioluminescence images, tumor ovaries images, and average abdominal circumference changes of ID8 tumor‐bearing mice (*n* = 6). The statistical significance of the difference was evaluated by two‐tailed Student's *t*‐test (two groups) or one‐way ANOVA with Tukey's post‐hoc test (three or more groups). Survival analysis was performed using the log‐rank (Mantel–Cox) test. ns means not significant, *p < 0.05, **p < 0.01, *** *p* < 0.001, **^#^** *p* < 0.05, **^###^** *p* < 0.001.

## Conflict of interest

The authors declare no conflict of interest.

## Supporting information

Supporting Information

## Data Availability

The data that support the findings of this study are available in the supplementary material of this article.
