# Supplementary material for: Reshaping Intratumoral Mononuclear Phagocytes with Antibody‐Opsonized Immunometabolic Nanoparticles
Source: Adv Sci (Weinh). 2023 Oct 22;10(34):2303298. doi: 10.1002/advs.202303298 (PMC10700695; doi:10.1002/advs.202303298)
Supplement: Supplementary file 1 — Supporting Information [file ADVS-10-2303298-s001.pdf]

ADVANCED SCIENCE
Open Access

Supporting Information

for *Adv. Sci.*, DOI 10.1002/advs.202303298

Reshaping Intratumoral Mononuclear Phagocytes with Antibody-Opsonized Immunometabolic Nanoparticles

Chang Liu, Yanfeng Zhou, Daoxia Guo, Yan Huang, Xiaoyuan Ji, Qian Li, Nan Chen*, Chunhai Fan* and Haiyun Song**

Supplementary Information

Reshaping intratumoral mononuclear phagocytes with antibody-opsonized immunometabolic nanoparticles

Chang Liu,[‡] Yanfeng Zhou,[‡,*] Daoxia Guo,[‡] Yan Huang, Xiaoyuan Ji, Qian Li,
Nan Chen,[*] Chunhai Fan,[*] Haiyun Song[*]

*Emails: yfzhou@shsmu.edu.cn
nchen@shnu.edu.cn
fanchunhai@sjtu.edu.cn
songhaiyun@shsmu.edu.cn

This supplemental file includes:

Figures S1-S25

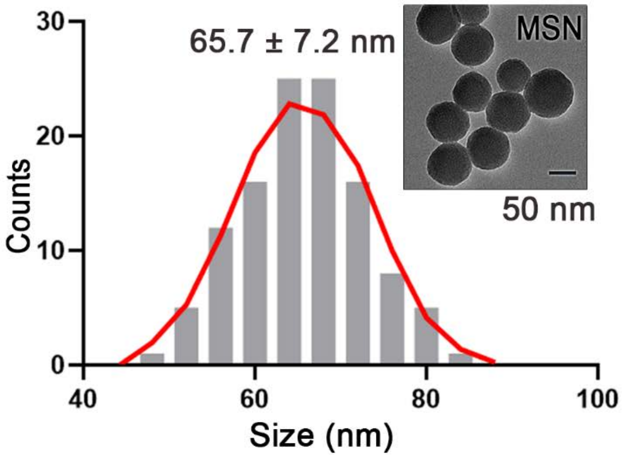

Figure S1. The size distribution of MSNs in TEM. Over 80 particles were counted. Insert: TEM image of MSNs.

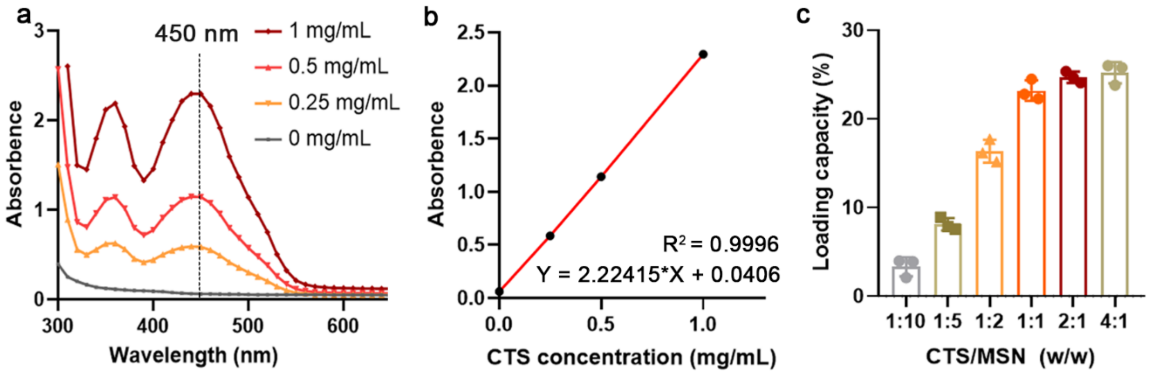

Figure S2. (a) UV-vis absorption spectra from CTS at various concentrations. (b) CTS standard curve. The absorbance of CTS at 450 nm was measured. A linear fit was applied to calculate the CTS concentration. (c) The loading efficiencies of CTS at various mass ratios of CTS to MSNs.

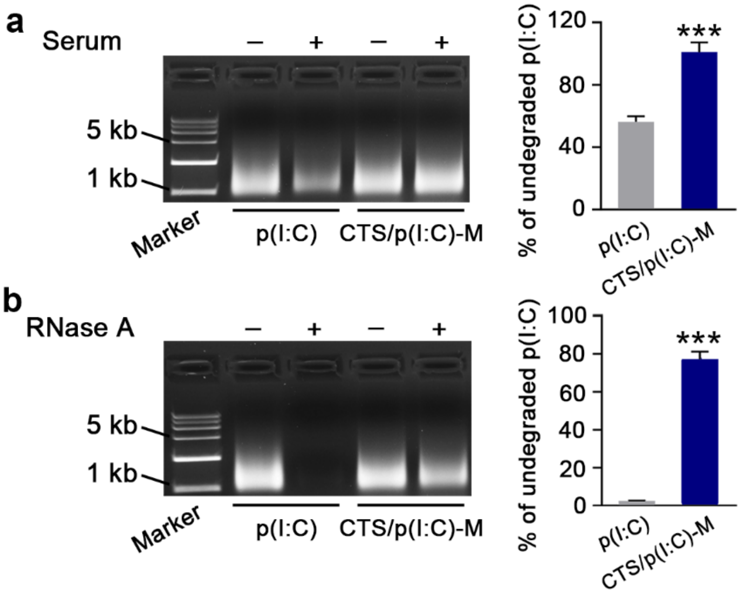

Figure S3. Stability of p(I:C) in CTS/p(I:C)-M. Left panels: agarose gel electrophoresis of free p(I:C) and CTS/p(I:C)-M before and after the treatment with 10% serum (a) or 1 mU RNase A (b). Right panels: quantification of undegraded p(I:C). Data are represented as mean ± SD (n = 3). Student's *t*-test, ***P <0.001.

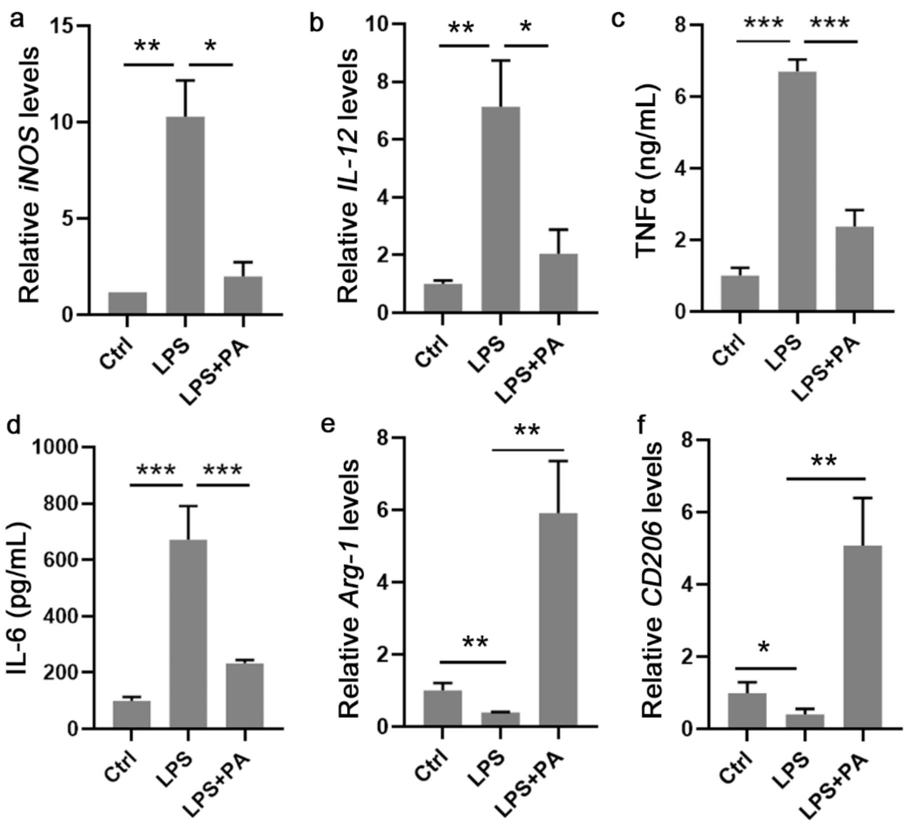

Figure S4. (a and b) RT-PCR analysis of M1 phenotype markers *iNOS* (a) and *IL-12* (b) expression in the BMDMs after indicated treatment. (c and d) The secretion of inflammatory cytokines TNF-α (c) and IL-6 (d) from the BMDMs after indicated treatment. (e and f) RT-PCR analysis of M2-type markers *Arg-1* (e) and *CD206* (f) expression in the BMDMs after indicated treatment. Data are represented as mean ± SD (n = 3). One-way ANOVA with Tukey's post-hoc test, ****P* < 0.001, ***P* < 0.01, **P* < 0.05.

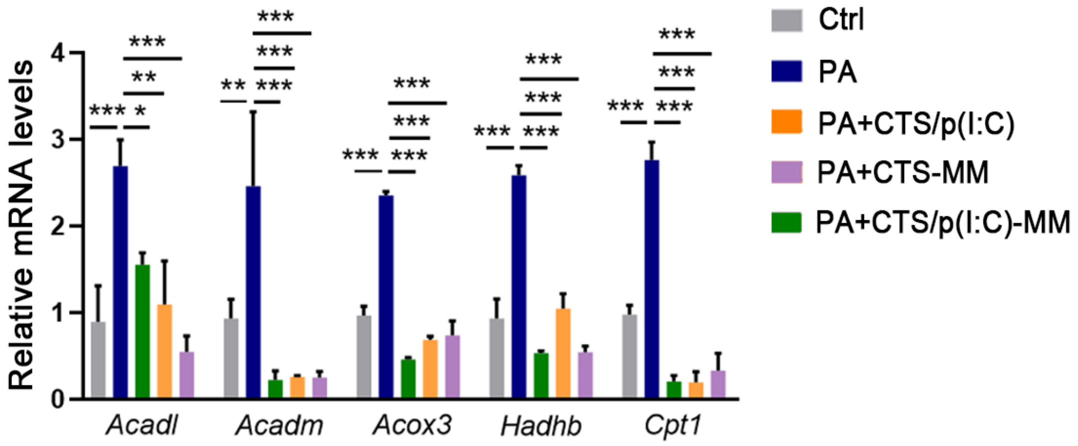

Figure S5. RT-PCR analysis of the FAO-related genes in LPS-induced BMDMs after indicated treatment. Data are represented as mean ± SD (n = 4). One-way ANOVA with Tukey's post-hoc test, ****P* < 0.001, ***P* < 0.01, **P* < 0.05.

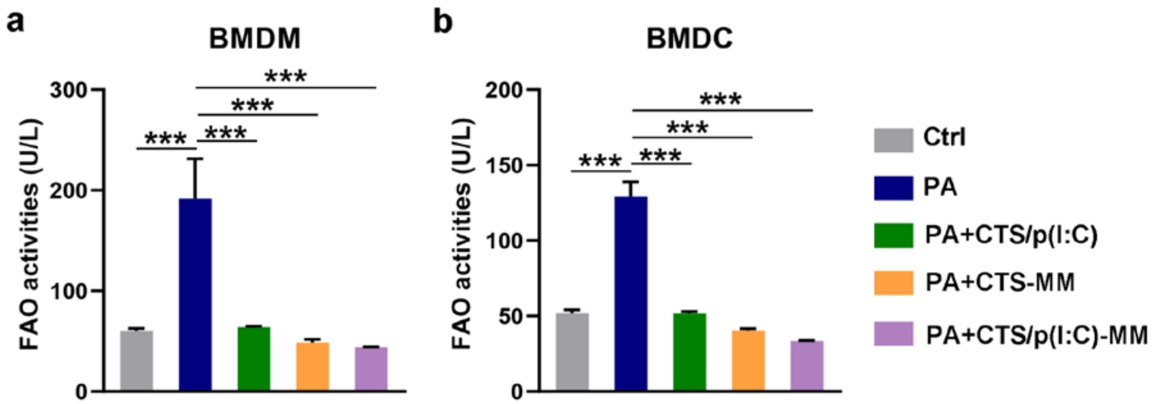

Figure S6. FAO activities in BMDMs (a) and BMDCs (b) after indicated treatment. Data are represented as mean ± SEM (n = 3). One-way ANOVA with Tukey's post-hoc test, ***P < 0.001.

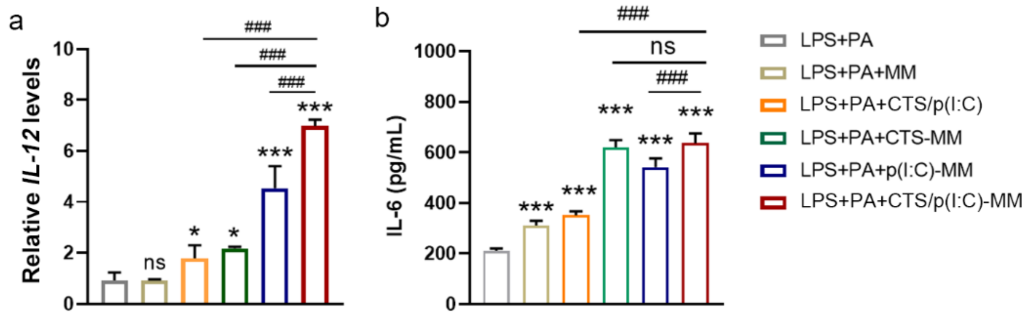

Figure S7. RT-PCR analysis of M1 phenotype marker *IL-12* (a) and the secretion of inflammatory cytokine IL-6 (b) in the BMDMs after indicated treatment. Data are represented as mean ± SD (n = 3). One-way ANOVA with Tukey's post-hoc test, ns means not significant, $^{***}P < 0.001$, $^{*}P < 0.05$, $^{###}P < 0.001$.

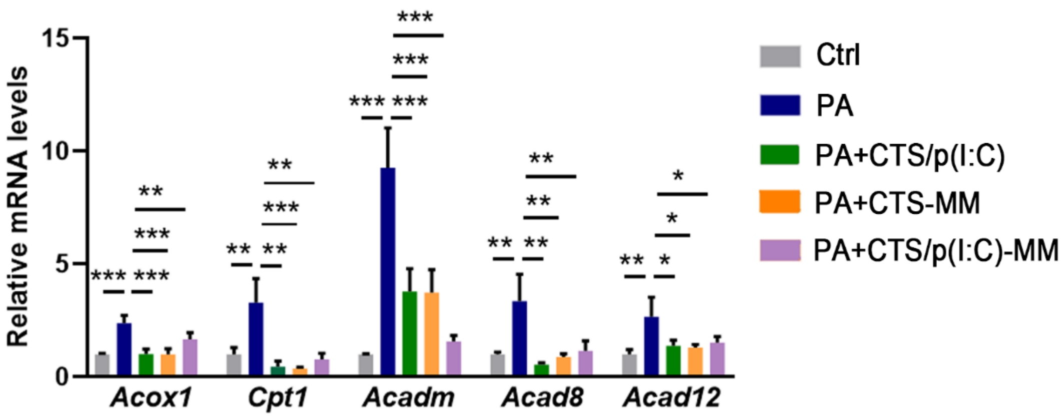

Figure S8. RT-PCR analysis of the FAO-related genes in LPS-induced BMDCs after indicated treatment. Data are represented as mean ± SD (n = 3). One-way ANOVA with Tukey's post-hoc test, ***$P < 0.001$, **$P < 0.01$, *$P < 0.05$.

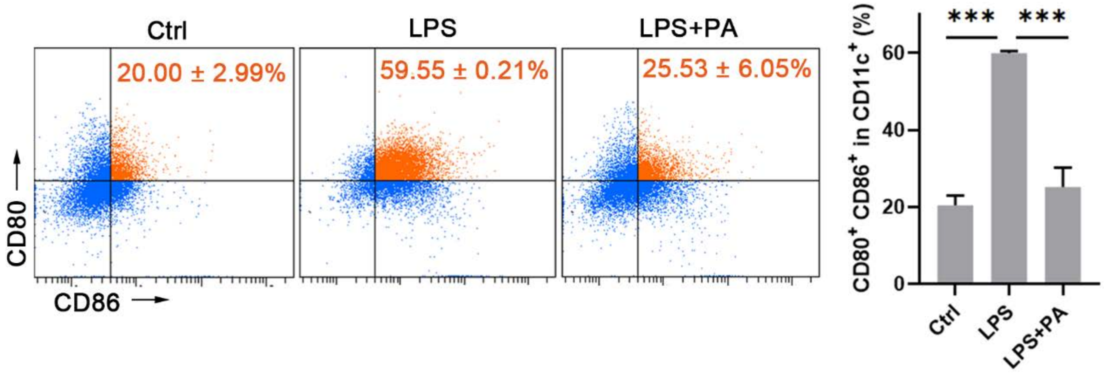

Figure S9. Flow cytometric analysis of $CD80^{+}CD86^{+}$ cells in BMDCs treated with LPS or LPS+PA. Data are represented as mean ± SD (n = 4). One-way ANOVA with Tukey's post-hoc test, ****P* < 0.001.

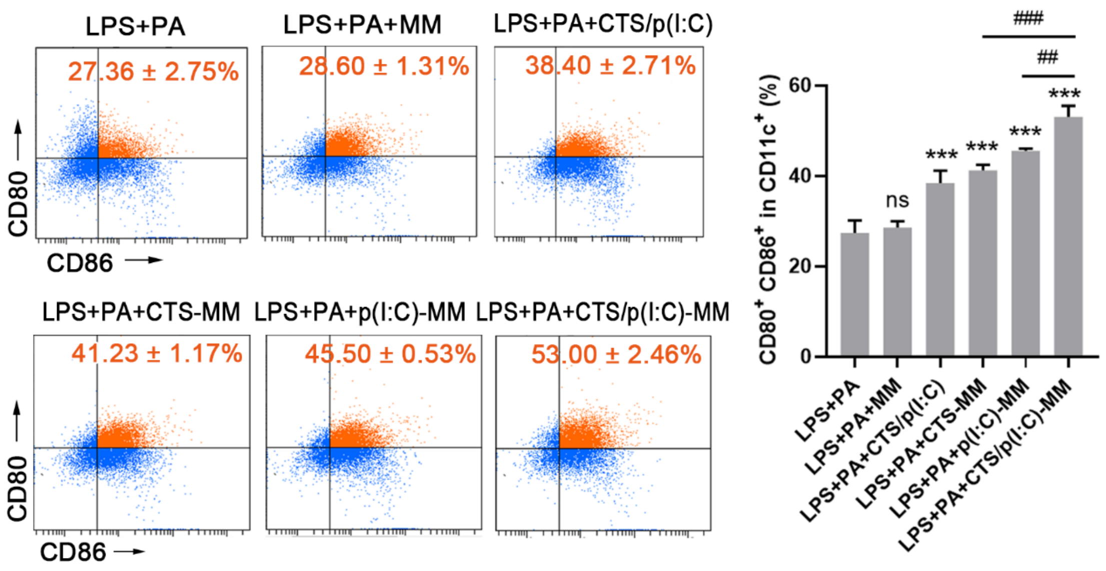

Figure S10. Flow cytometric analysis of $CD80^{+}CD86^{+}$ cells in BMDCs after indicated treatment. Data are represented as mean ± SD (n = 3). One-way ANOVA with Tukey's post-hoc test, ns means not significant, $^{***}P < 0.001$, $^{###}P < 0.001$, $^{##}P < 0.01$.

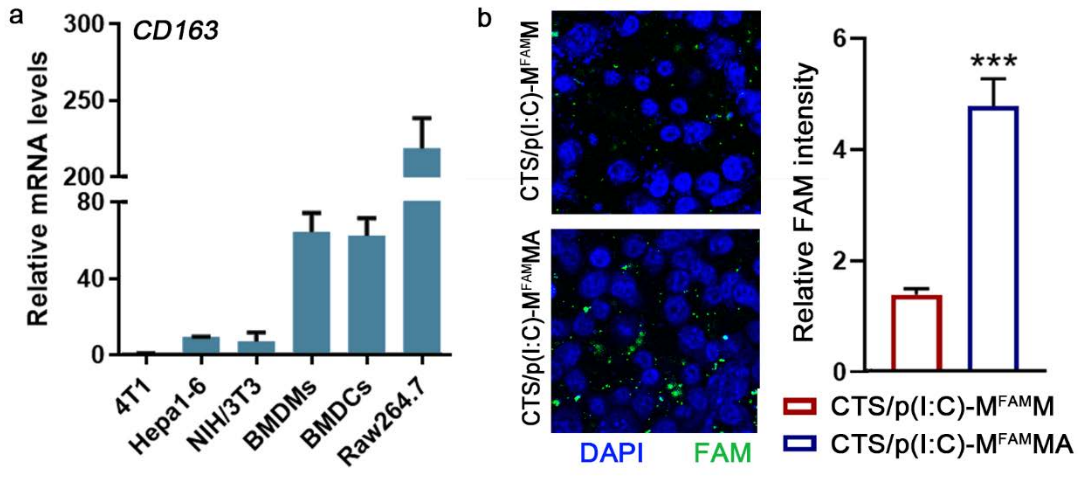

Figure S11. (a) RT-PCR analysis of *CD163* expression in cancer cells (4T1 and Hepa1-6), fibroblasts (NIH/3T3), and mononuclear phagocytes (BMDMs, BMDCs and Raw 264.7). (b) Cellular uptake efficiency of different forms of immeNPs (labeled by FAM, green) in Raw 264.7 macrophages. Data are represented as mean ± SD (n = 3). Student's *t*-test, ***$P < 0.001$.

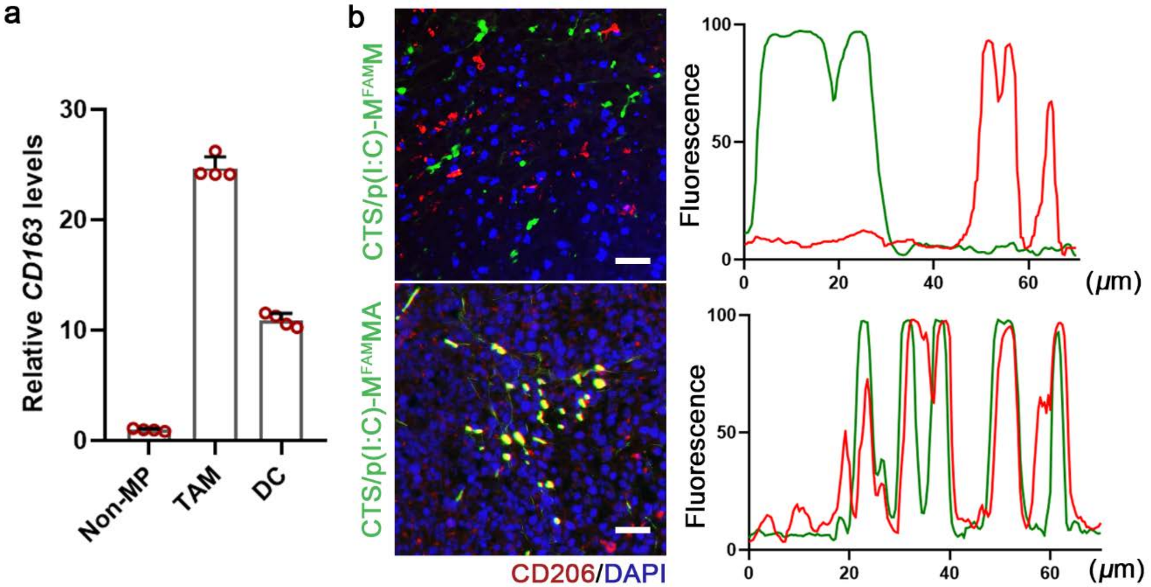

Figure S12. (a) RT-PCR analysis of *CD163* expression in TAMs, DCs and non-MPs from 4T1 tumors. Data are represented as mean ± SD (n = 4). (b) The co-localization of different forms of immeNPs (labeled by FAM, green) with the TAMs (labeled by anti-CD206, red) in 4T1 tumor sections from inoculated mice. Scale bars, 30 μm.

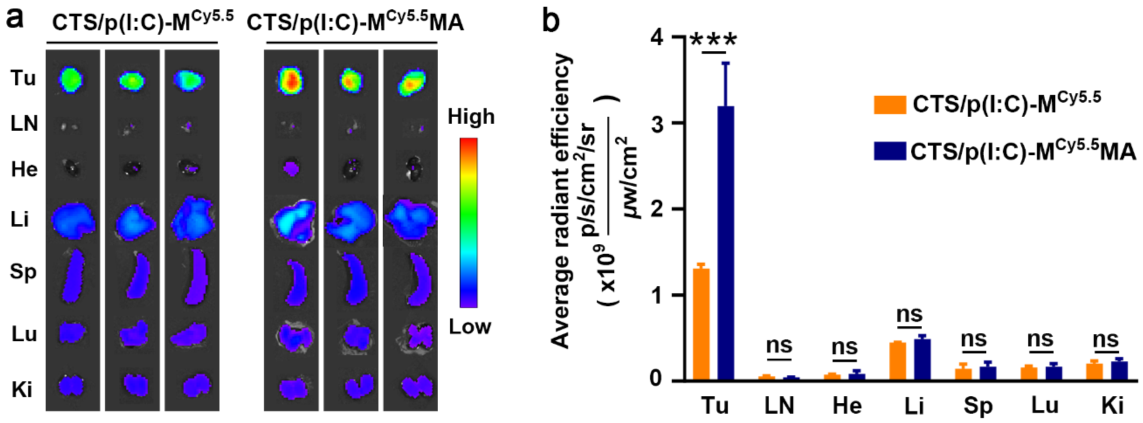

Figure S13. Tissue distribution of the immeNPs. (a) The *ex-vivo* fluorescence imaging of major organs and tumors after intravenous injection of CTS/p(I:C)-$M^{Cy5.5}$ and CTS/p(I:C)-$M^{Cy5.5}$MA. (b) Quantitative analysis of the fluorescent intensities of major organs and tumors in panel (a). Data are represented as mean ± SD (n = 3). Student's *t*-test, ns means not significant, ***$P < 0.001$.

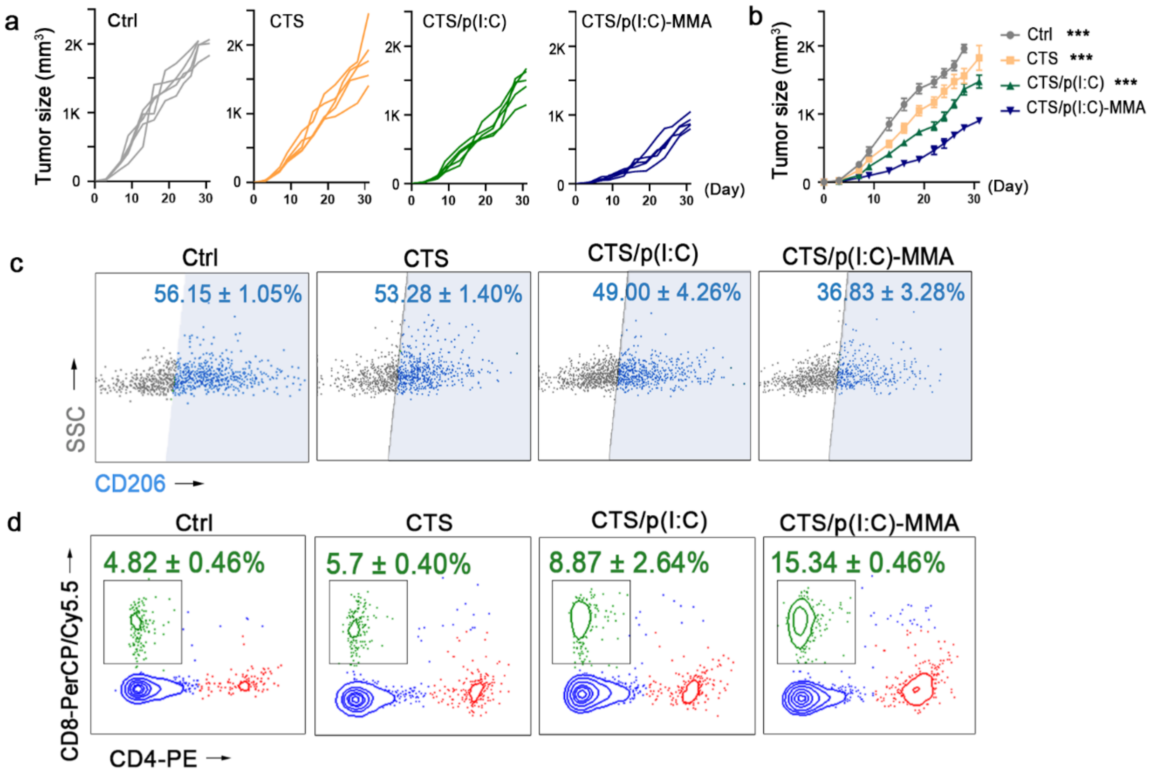

Figure S14. Therapeutic efficacies of immeNPs in 4T1 TNBC model. (a) Individual tumor growth kinetics (n = 6). (b) Average tumor growth curves (n = 6). (c) Flow cytometric analysis of the percentage of M2-TAM population in TAMs (gated on $CD45^+CD11b^+F4/80^+$) (n = 3). (d) Flow cytometric analysis of the percentage of $CD8^+$ T cells in total immune cells (gated on $CD45^+$ cells) within tumors (n = 3). Data are represented as mean ± SD in (b-d). One-way ANOVA with Tukey's post-hoc test, ***$P < 0.001$.

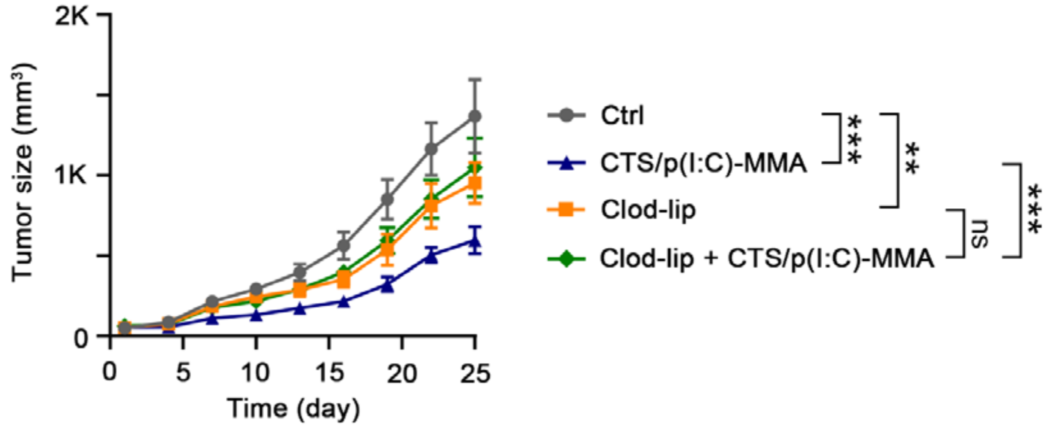

Figure S15. Average 4T1 tumor growth curves after intravenous injection of CTS/p(I:C)-MMA in the absence or presence of clodronate disodium liposomes. Data are represented as mean ± SD (n = 7). One-way ANOVA with Tukey's post-hoc test, ns means not significant, ***P < 0.001, **P < 0.01.

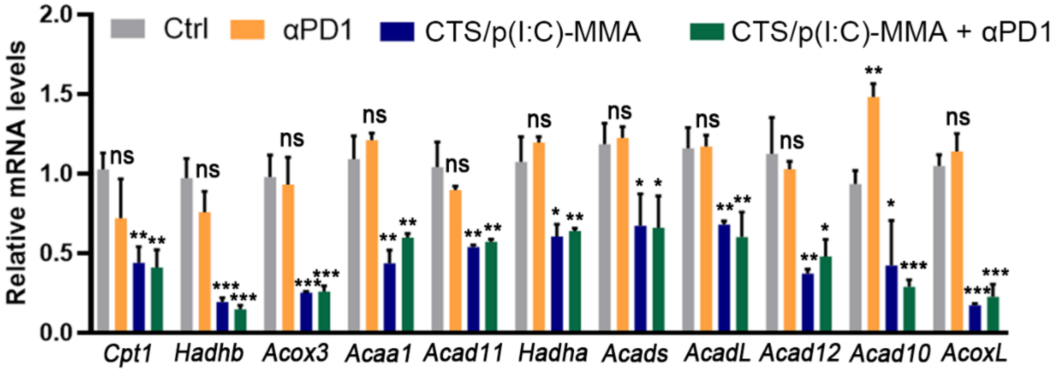

Figure S16. RT-PCR analysis of the FAO-related genes. Data are represented as mean ± SD (n = 3). One-way ANOVA with Tukey's post-hoc test, ns means not significant, ***P < 0.001, **P < 0.01, *P < 0.05.

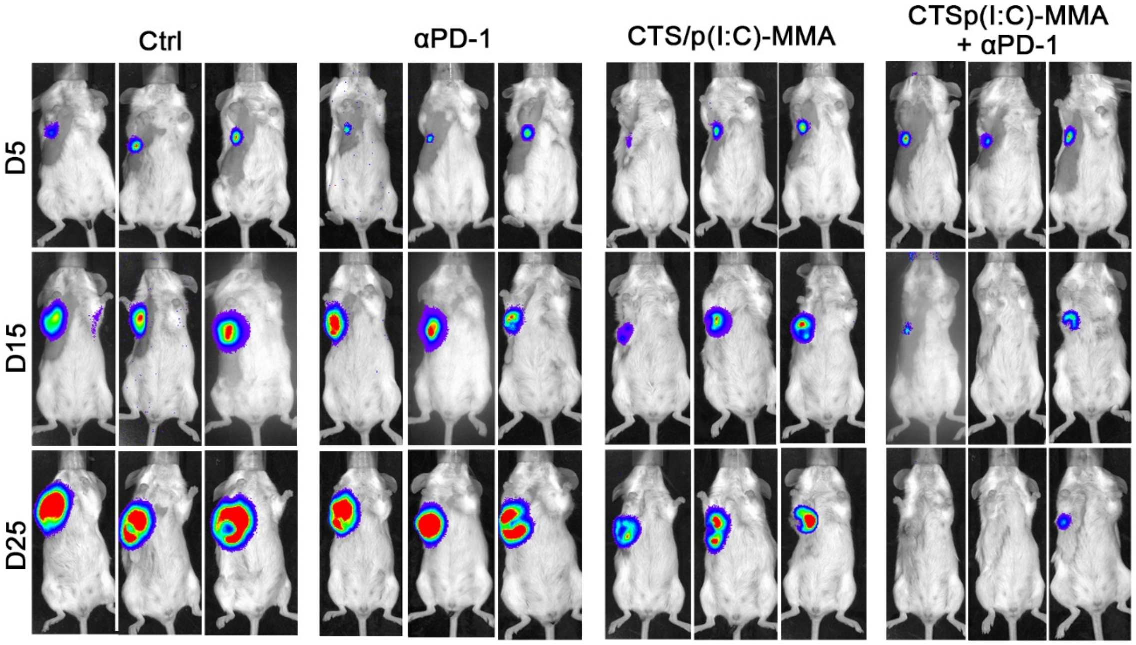

Figure S17. The *in-vivo* bioluminescence imaging of 4T1 tumor-bearing mice at various time points.

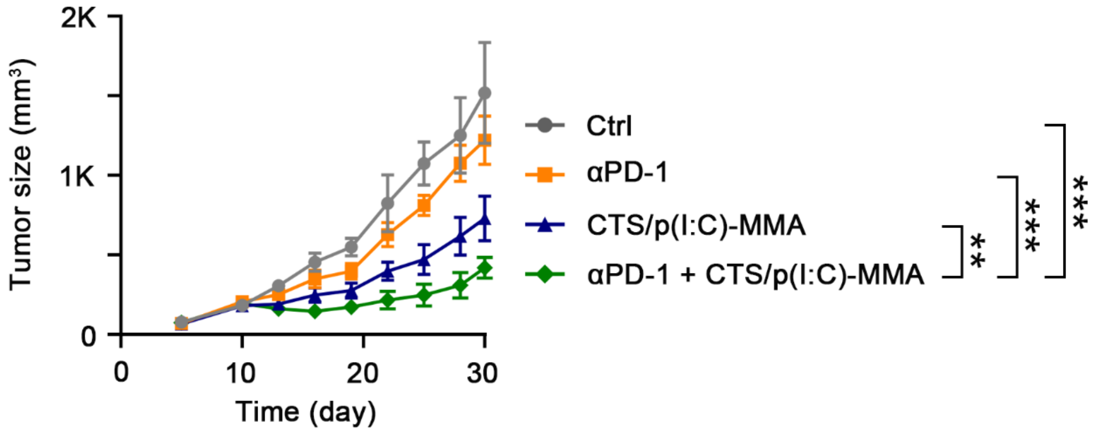

Figure S18. Average tumor growth curves of indicated groups. The CTS/p(I:C)-MMA treatment started at 10 days after tumor cell inoculation. Data are represented as mean ± SD (n = 7). One-way ANOVA with Tukey's post-hoc test, ***$P < 0.001$, **$P < 0.01$.

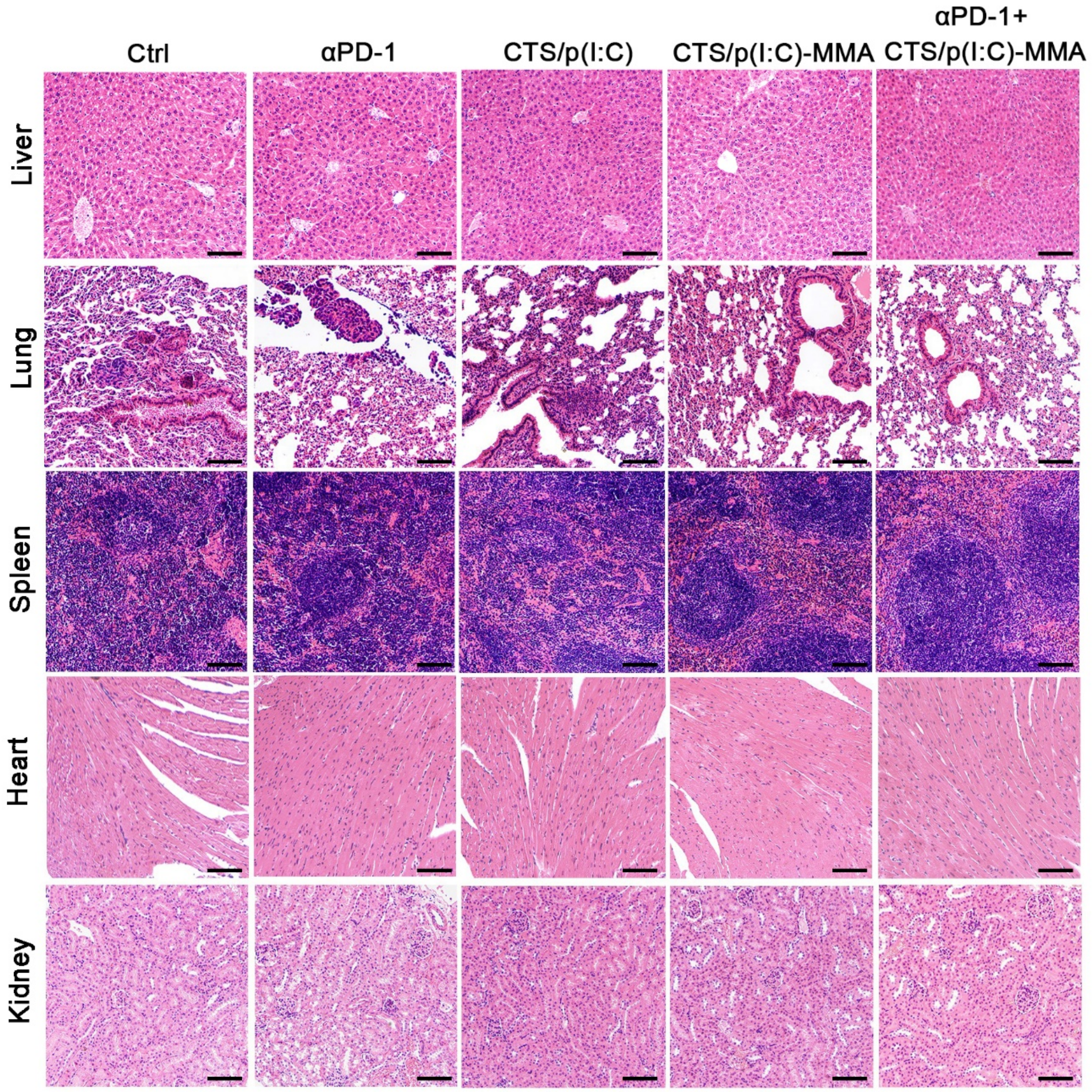

Figure S19. H&E staining of major organs collected from indicated groups. Scale bars = 100 μm.

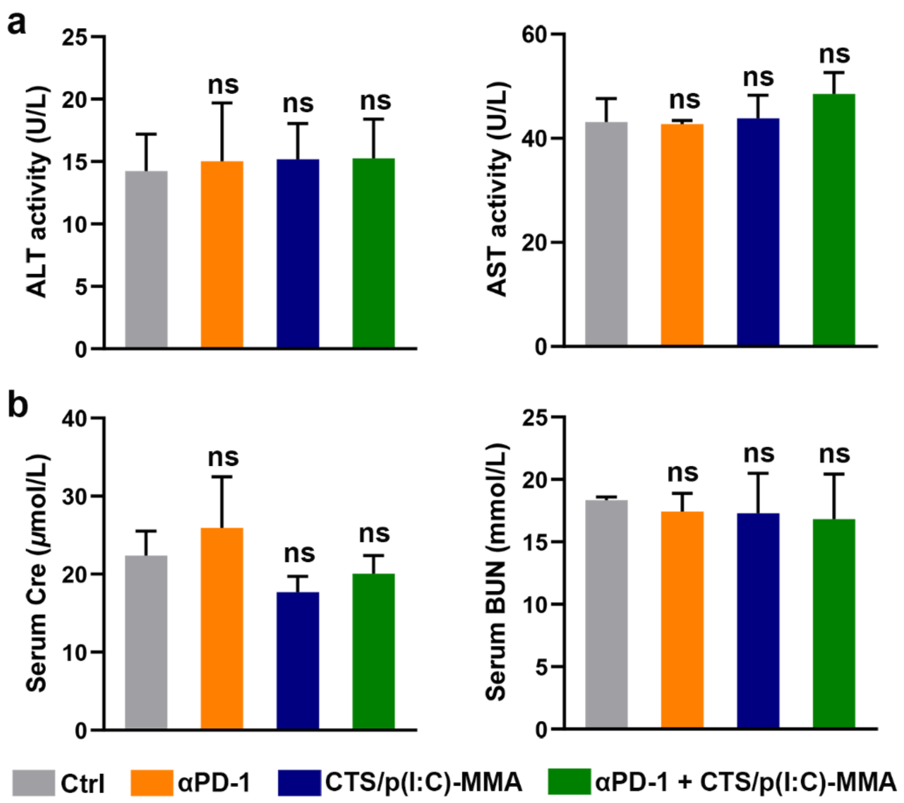

Figure S20. Effects of immeNPs on serum levels of liver and kidney function parameters. (a) Serum alanine transaminase (ALT) and aspartate transaminase (AST) activities in tumor-bearing mice after indicated treatment. (b) Serum levels of creatinine (Cre) and blood urea nitrogen (BUN) in tumor-bearing mice after indicated treatment. Data are represented as mean ± SEM (n = 3). One-way ANOVA with Tukey's post-hoc test, ns means not significant.

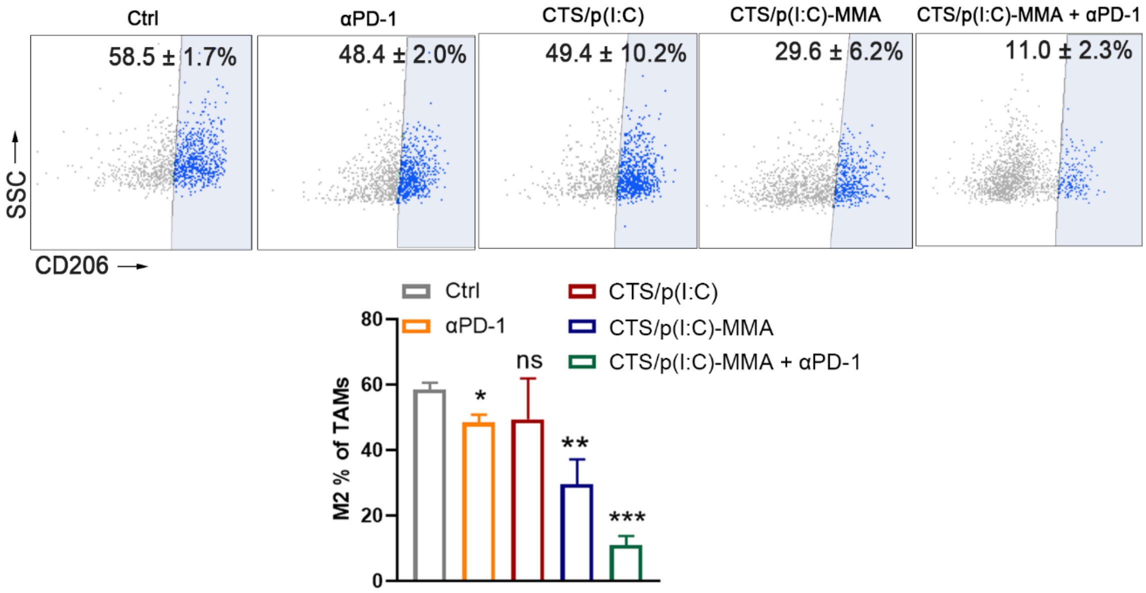

Figure S21. Flow cytometric analysis of the percentage of M2-TAM population in TAMs (gated on $CD45^+CD11b^+F4/80^+$). Data are represented as mean ± SD (n = 3). One-way ANOVA with Tukey's post-hoc test, ns means not significant, ***$P < 0.001$, **$P < 0.01$, *$P < 0.05$.

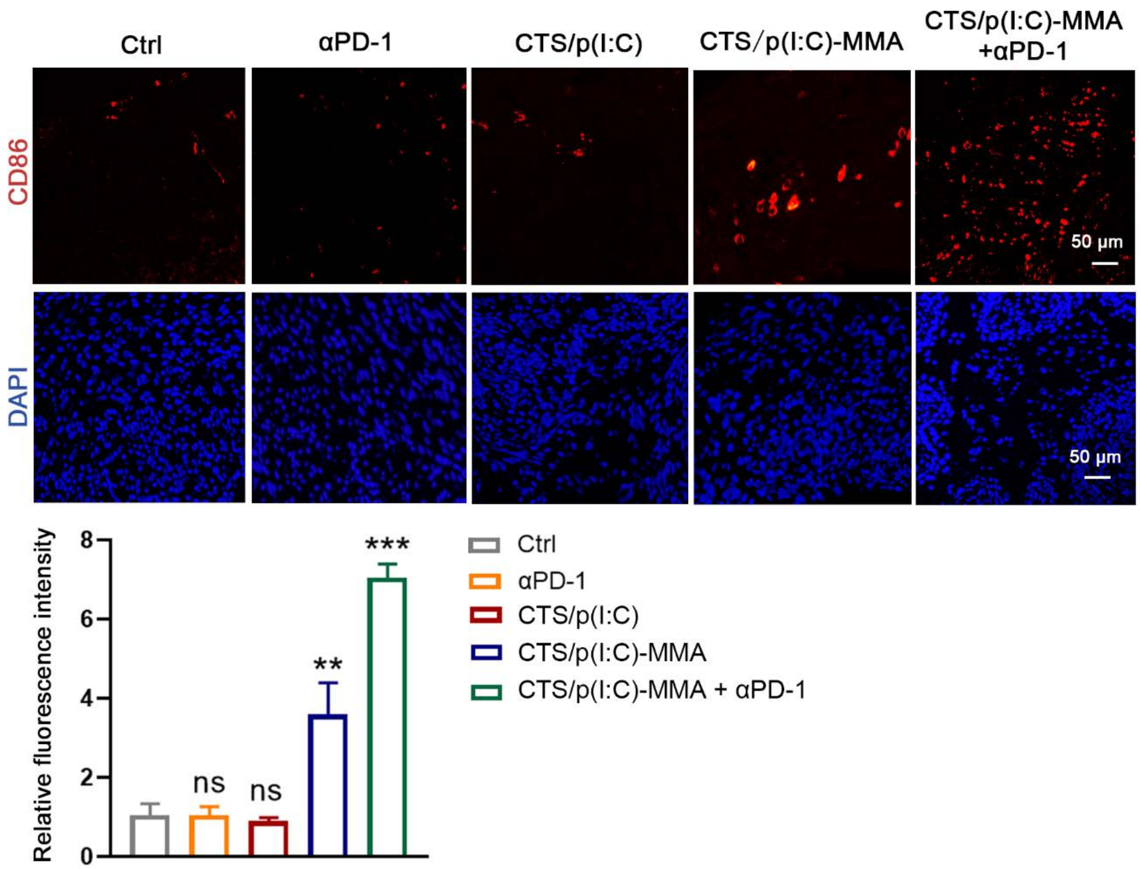

Figure S22. Representative immunofluorescence images and quantitative analysis of $CD86^+$ DCs in ID8 tumor sections. Scale bars, 50 μm. Data are represented as mean ± SD (n = 3). One-way ANOVA with Tukey's post-hoc test, ns means not significant, ***P < 0.001, **P < 0.01.

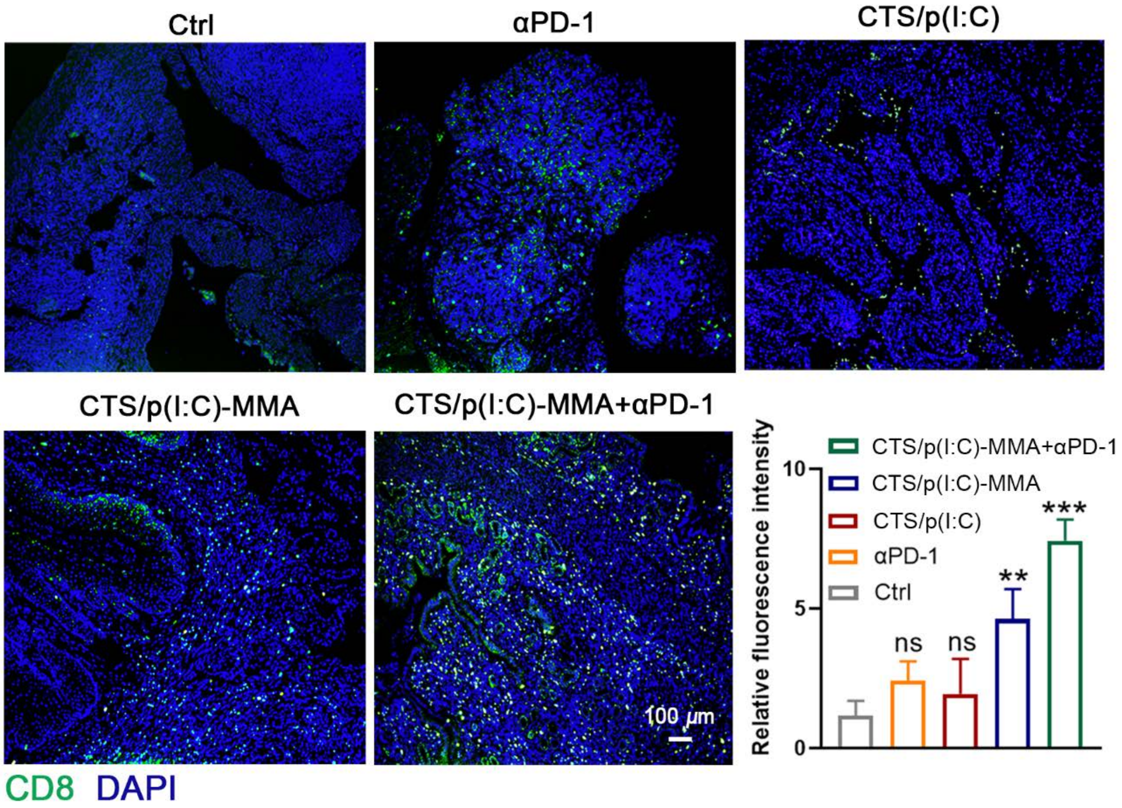

Figure S23. Representative immunofluorescence images and quantitative analysis of $CD8^+$ T cells in ID8 tumor sections. Scale bars, 100 μm. Data are represented as mean ± SD (n = 4). One-way ANOVA with Tukey's post-hoc test, ns means not significant, ***$P < 0.001$, **$P < 0.01$.

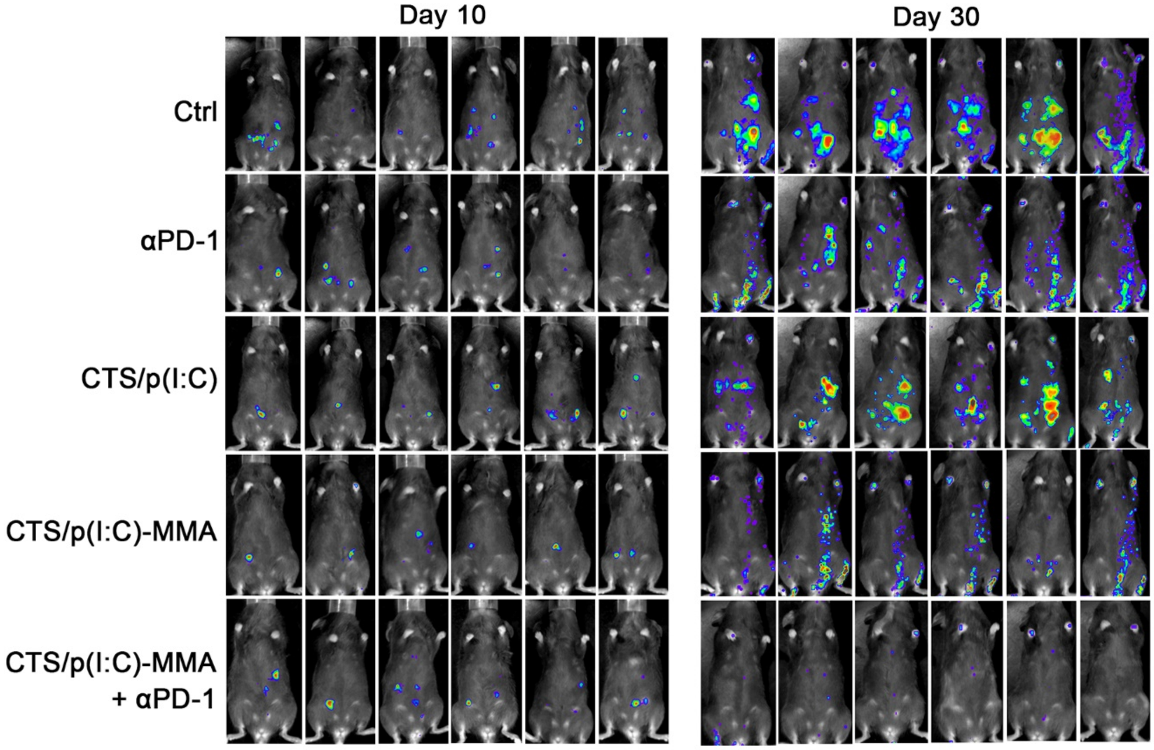

Figure S24. The *in-vivo* bioluminescence imaging of ID8 tumor-bearing mice at various time points.

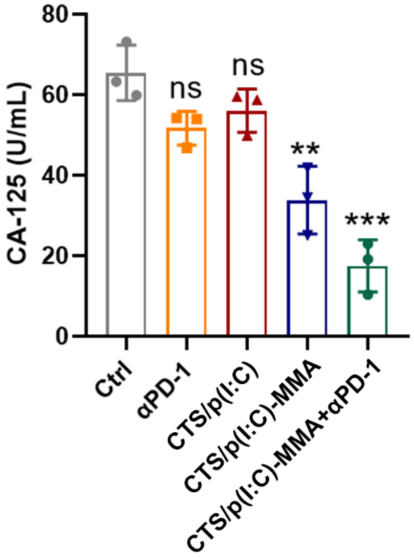

Figure S25. The serum levels of ovarian cancer biomarker CA-125 in ID8 tumor-bearing mice after indicated treatment. Data are represented as mean ± SD (n = 3). One-way ANOVA with Tukey's post-hoc test, ns means not significant, ***$P < 0.001$, **$P < 0.01$.
